# A new case of Rafiq syndrome with coexisting thyroid dyshormonogenesis type 6 in a Chinese patient: case report and literature review

**DOI:** 10.3389/fendo.2025.1583011

**Published:** 2025-11-27

**Authors:** Ruifang Qi, Hanfei Guo, Rongmin Li, Jie Chang, Di Wu, Yanmei Sang

**Affiliations:** 1 Department of Pediatric Endocrinology, Baoding Hospital of Beijing Children's Hospital Affiliated to Capital Medical University, Baoding, China; 2 Department of Pediatric Endocrinology, Genetic and Metabolism, Beijing Children’s Hospital, Capital Medical University, National Center for Children’s Health, Beijing, China

**Keywords:** Rafiq syndrome, MAN1B1 gene mutation, *de novo* mutation, thyroid dyshormonogenesis type 6, China

## Abstract

Rafiq syndrome is a rare autosomal recessive genetic disorder first described by Rafiq et al. in 2011.With an extremely low incidence rate, just over 40 cases have been reported worldwide. This condition is caused by mutations in the MAN1B1 gene, which encodes a member of the glycosyl hydrolase family 47.The primary clinical features of Rafiq syndrome include intellectual and motor developmental delay, distinctive facial features, truncal obesity, and hypotonia. We described a case of Rafiq syndrome with coexisting thyroid dyshormonogenesis type 6 in a Chinese Patient. The patient presented with distinctive facial features (small forehead, wide eye distance, small bilateral eye fissures, low nose bridge, protruding nose, short philtrum, small chin, large ears, short neck), borderline intellectual delay, truncal obesity, abnormal coagulation function, abnormal electroencephalogram, which were similar with the clinical manifestations of Rafiq syndrome reported in the literature. In addition to the above-mentioned abnormalities, the child also has thyroid dyshormonogenesis type 6. Genetic testing has identified compound heterozygous mutations in the MAN1B1 gene: c.1281_1303delCATCCACGCCTGTGTCTGGAAGA and c.2011C>T, and one heterozygous mutation in the DUOX2 gene: c.650A>G, which is new variant of uncertain clinical significance. The clinical manifestations and genetic testing of patients can help diagnose Rafiq syndrome. To the best of our knowledge, this combination of genetic defects is unique and has not been previously reported in the literature.

## 1 Introduction

Rafiq syndrome is a rare autosomal recessive genetic disorder first described by Rafiq et al. in 2011 (1). With an extremely low incidence rate, just over 40 cases have been reported worldwide (2). This condition is caused by mutations in the MAN1B1 gene, which encodes a member of the glycosyl hydrolase family 47. The primary clinical features of Rafiq syndrome include intellectual and motor developmental delay, distinctive facial features, truncal obesity, and hypotonia. The distinctive facial features include small forehead, wide eye distance, small bilateral eye fissures, low nose bridge, protruding nose, short philtrum, small chin, large ears, short neck (1).

The diagnosis of Rafiq syndrome can be confirmed through clinical evaluation and genetic testing. However, due to its rarity, the condition is often overlooked or misdiagnosed in clinical practice. The increasing application of genetic analysis in clinical practice will facilitate the diagnosis of this rare syndrome without the need for recognition of detailed clinical sequalae in some countries, whereas in others this would still be an important step toward the proper diagnosis. This article provides a detailed summary and analysis of a genetically confirmed case of Rafiq syndrome combined with thyroid dyshormonogenesis (TDH) type 6 in a Chinese pediatric patient. The aim is to enhance clinicians’ understanding of this rare disorder and improve diagnostic accuracy.

## 2 Clinical data

**Figure 1:**
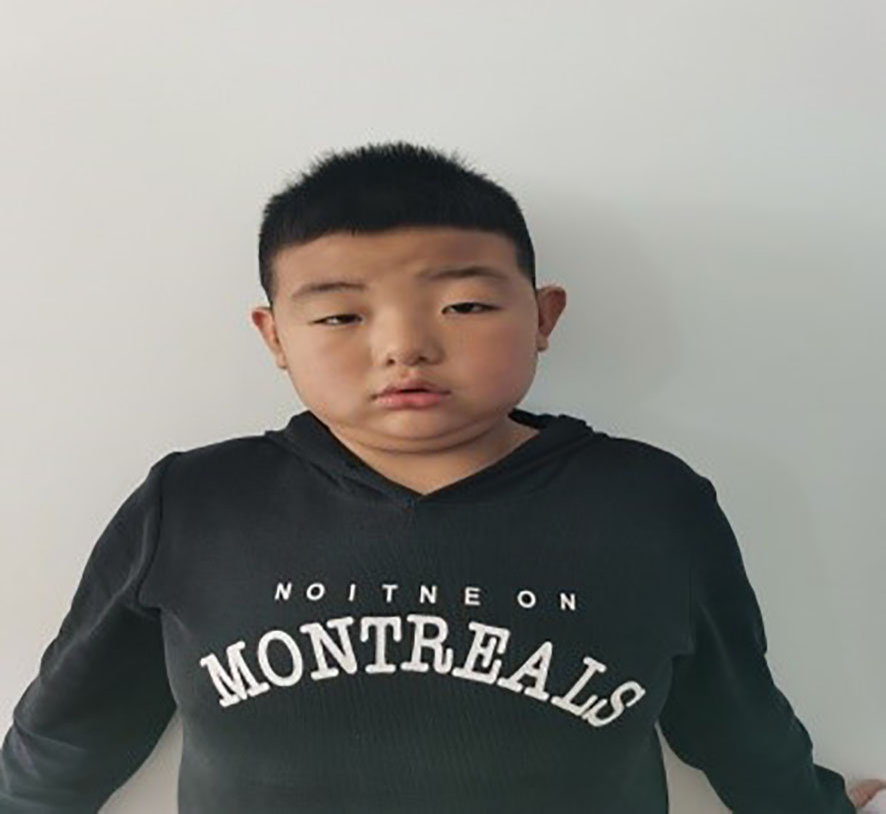
Note the small forehead, wide eye distance, small bilateral eye fissures, low nose bridge, protruding nose, short philtrum, small chin, mentioned in the text.

**Figure 2:**
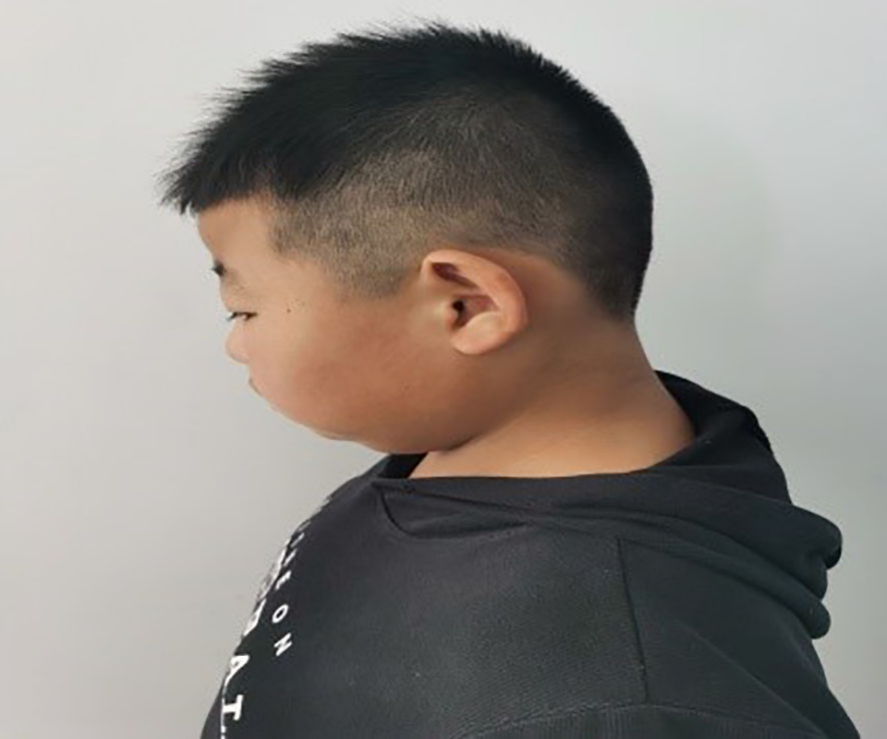
Note the big ears in the text.

**Figure 3:**
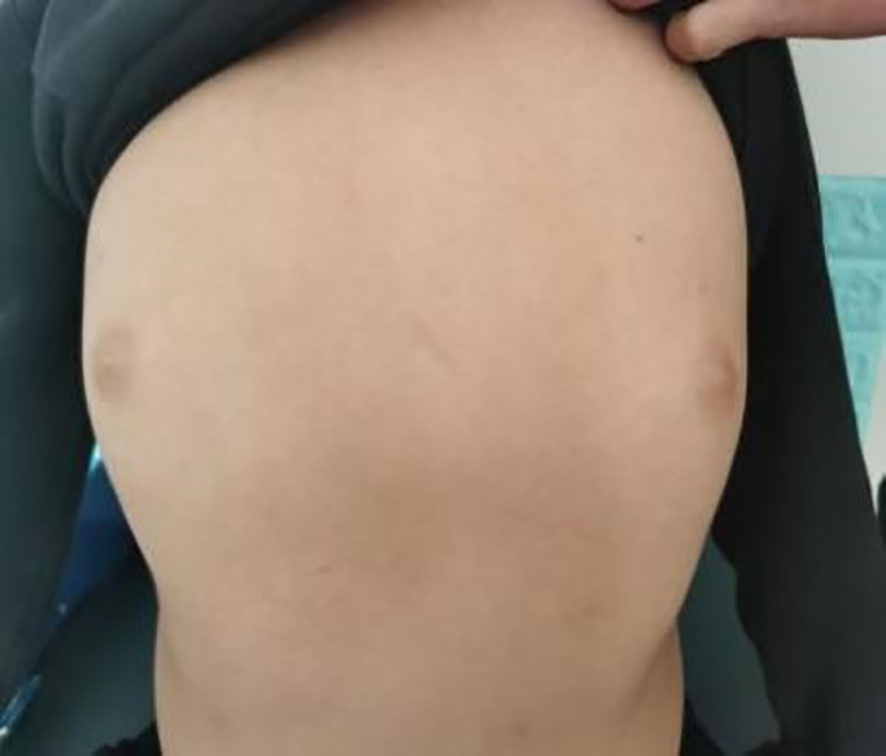
Note the obese habitus in the text.

The patient, a 9-year-old male, was brought to our endocrinology clinic in June 2021 due to a history of congenital hypothyroidism (CH) and intellectual disability. He was born to a G2P2 mother via cesarean section at 38 weeks and 5 days, with a birth weight of 3,150 g and a birth length of 50 cm. His mother underwent routine prenatal checkups during pregnancy, with no significant abnormalities detected. Postnatal feeding was normal. At 2 months of age, he was diagnosed with CH based on abnormal heel-prick blood test results and received oral treatment: LT4:4.4μg/kg/d, taken in the morning and checked regularly for five thyroid functions. Currently, LT4: dose is 1.5μg/kg/d, taken in the morning. The child learned to walk and have simple conversations at the age of 1 year and 3 months. At the age of 3, the child showed intellectual disability, poor comprehension, poor expression ability, and poor learning. However, the child’s height increased normally every year and their weight increased by 10kg per year. Physical examination on admission showed that his weight was 50 kg (>95th percentile), height was 138.5 cm (50th percentile), and his BMI was 26.2 kg/m² (>95th percentile). The child has an obese body shape and can see special facial features, including small forehead, wide eye distance, small bilateral eye slits, low nose bridge, protruding nose, short stature, small chin, large ears, short neck, etc (**Figures 1**–**3**). No abnormalities were found in the muscle strength and tension of the child’s limbs. The boy’s external genitalia showed no abnormalities.

Diagnostic testing revealed that the thyroid function panel (five parameters) conducted at 3 months of age showed total triiodothyronine (TT3):4.1 nmol/L (normal: 1.84-3.24 nmol/L), free triiodothyronine (FT3):11.35 pmol/L (normal: 4.5-7.3 pmol/L), total thyroxine (TT4):280nmol/L (normal:82.3-167.0 nmol/L), free thyroxine (FT4): 35 pmol/L (normal:10.8–20 pmol/L), and thyroid-stimulating hormone (TSH): >100 mIU/L (normal:0.8-5.0 mIU/L). The FT3 and FT4 values of the patient are higher than the normal range, which is considered to be related to the supplementation of LT4. However, the TSH of the patient is still significantly higher than normal, so the LT4 dose is adjusted to 33ug/time and taken in the morning for treatment. The panel repeated at 5 months of age showed TT3:2.86 nmol/L (normal: 1.84-3.24nmol/L), FT3:7.35 pmol/L (normal: 4.5-7.3 pmol/L), TT4:188.1 nmol/L (normal: 82.3-167.0 nmol/L), FT4 of 22.6 pmol/L (normal: 10.8–20 pmol/L), and TSH of 7.005 mIU/L (normal: 0.8-5.0 mIU/L). At 9 years of age, the thyroid function panel demonstrated values within the reference range (See **Table 1** for details).

**Table 1:** The thyroid function panel (five parameters).

Age	TT3 (normal 1.84-3.24)	FT3 (normal 4.5-7.3)	TT4 (normal 82.3-167)	FT4 (normal 10.8-20)	TSH (normal 0.8-5.0)	W	LT4
2 months	–	–	–	–	>100	4.5kg	4.4μg/kg/d
3 months	4.1	11.35	280	35	>100	5.8kg	5.6μg/kg/d
5 months	2.86	7.35	188.1	22.6	7.005	8kg	3.1μg/kg/d
6 months	3.2	5.87	154	15	2.22	8.4kg	2.9μg/kg/d
3 years	2.8	6.5	135	13	59.72	17kg	2.9μg/kg/d
9 years	3.1	5.9	142	17	2.86	50kg	1.5μg/kg/d

Comprehensive biochemical analysis and sex hormone panel (six parameters) were within normal limits. Chromosomal karyotyping confirmed a 46XY karyotype. The thyroid autoantibody panel (four parameters) was normal. The glycated hemoglobin (HbA1c) level was 5.9% (normal: 4.0-6.0%), while the 25-hydroxy vitamin D level was 14.4 μg/L (normal: ≥ 30 μ g/L). The blood insulin level was 15.5 μIU/mL (normal: 6-27uIU/mL).

Color Doppler echocardiogram demonstrated normal chamber dimensions and cardiac structure. Abdominal color Doppler ultrasound identified hepatic steatosis, whereas the gallbladder, pancreas, spleen, and kidneys appeared normal. Thyroid color Doppler ultrasound did not detect normal thyroid tissue in the expected thyroid anatomical location. Instead, only a hypoechoic nodule (1.3 × 1.1 × 1.2 cm) was detected at the base of the tongue, suggesting ectopic thyroid tissue. We conducted an intelligence test for the child, and the score was 76 points. According to the Stanford–Binet Fifth Edition (SB5) classification, an IQ of 76 is classified as borderline delay. Urine screening showed mild ketotic dicarboxylic aciduria, with slightly elevated succinate levels, this may have been randomly discovered. Brain magnetic resonance imaging (MRI) revealed no abnormalities, while electroencephalography (EEG) showed an abnormal pediatric pattern, with spike-and-wave discharges in the bilateral occipital and left posterior temporal regions during sleep. The coagulation panel (seven parameters) demonstrated a plasma antithrombin III level of 68% (Normal: 83-128%).

## 3 Genetic analysis

All clinical investigations and therapeutic interventions were performed with informed consent from the patient’s legal guardians and were approved by the institutional ethics committee.

After obtaining informed consents, 3 mL of EDTA-anticoagulated peripheral blood was collected from the patient and his parents. High-throughput whole exome sequencing (WES) was performed by Beijing Maikeno Technology Co., Ltd.

Briefly, genomic DNA was extracted from the blood samples and subjected to DNA library preparation. The library was then hybridized with biotinylated probes targeting the whole exome (P039-Exom, Maikeno) under optimized conditions. Streptavidin-coated magnetic beads were utilized to capture the biotinylated probe-target DNA complexes. The captured DNA fragments were then eluted, purified, and enriched. The enriched target DNA was sequenced using the Illumina NextSeq 500 platform. Sequencing reads were aligned to the human reference genome hg19 using the Burrows-Wheeler Aligner (BWA) software. The resulting alignment files were sorted, filtered, and locally realigned to reduce false-positive variant calls. Variants of potential clinical significance were confirmed by Sanger sequencing.

### 3.1 Genetic sequencing results

**Figure 4:**
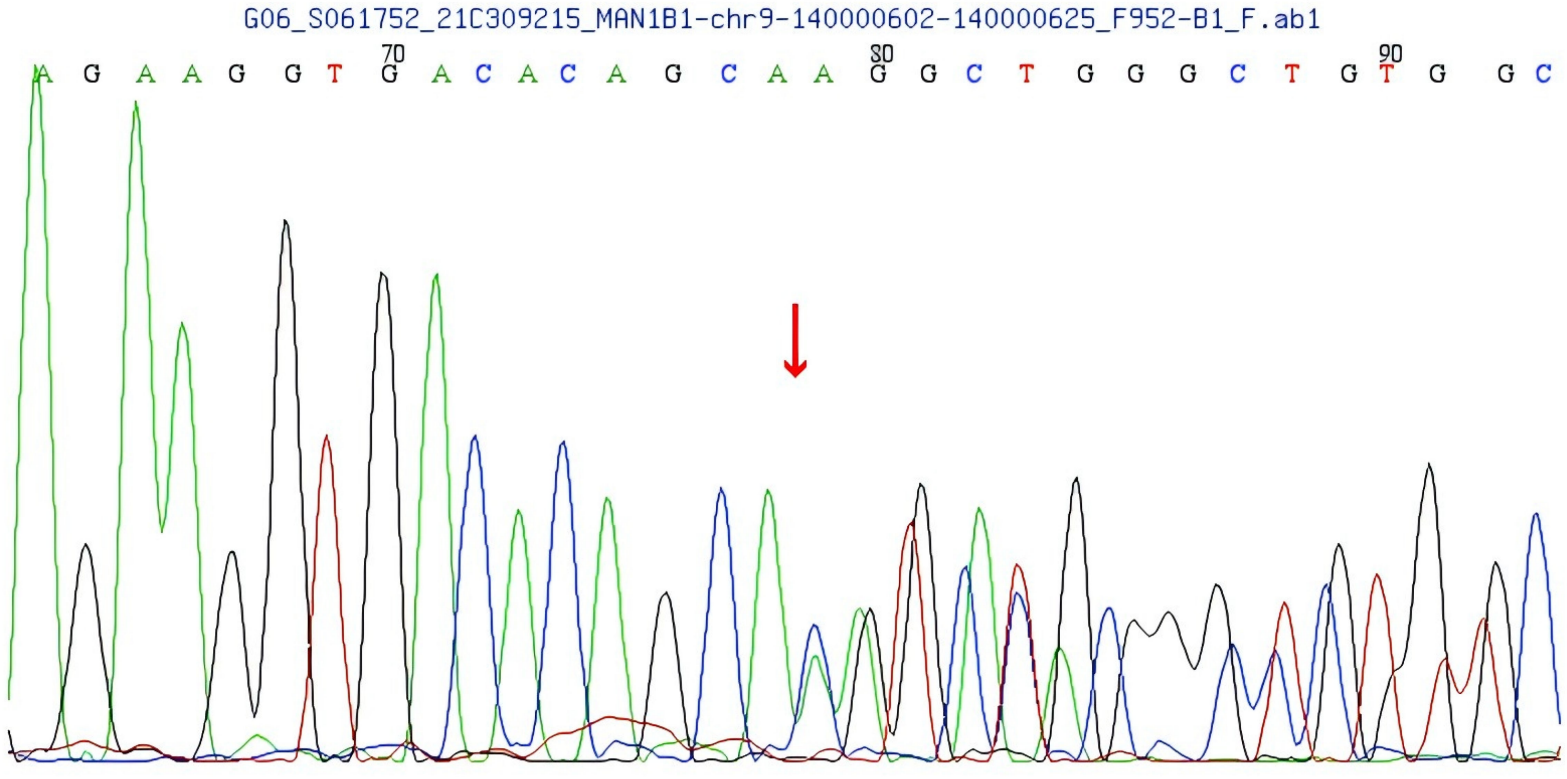
MAN1B1 gene frameshift variant c.1281_1303delCATCCACGCCTGTGTCTGGAAGA.

Compound heterozygous variants were identified in the MAN1B1 gene of the patient: c.1281_1303delCATCCACGGCCTGTCTGGGAAGA and c.2011C>T. The first one is a frameshift variant located in exon 9 of the MAN1B1 gene, c.1281_1303delCATCCACCGGCCTGTCTGTGGAAGA, resulting in an amino acid change of p.H427Qfs * 33 (See **Figure 4**). Family pedigree analysis confirmed paternal inheritance of this deletion, while the mother had a normal genotype at this locus. There are no correlation reports for this locus in the literature database, and there are no pathogenicity analysis results for this locus in the Clinvar database. According to the ACMG guidelines, this variant is preliminarily classified as a suspected pathogenic variant (PVS1+PM2).

**Figure 5:**
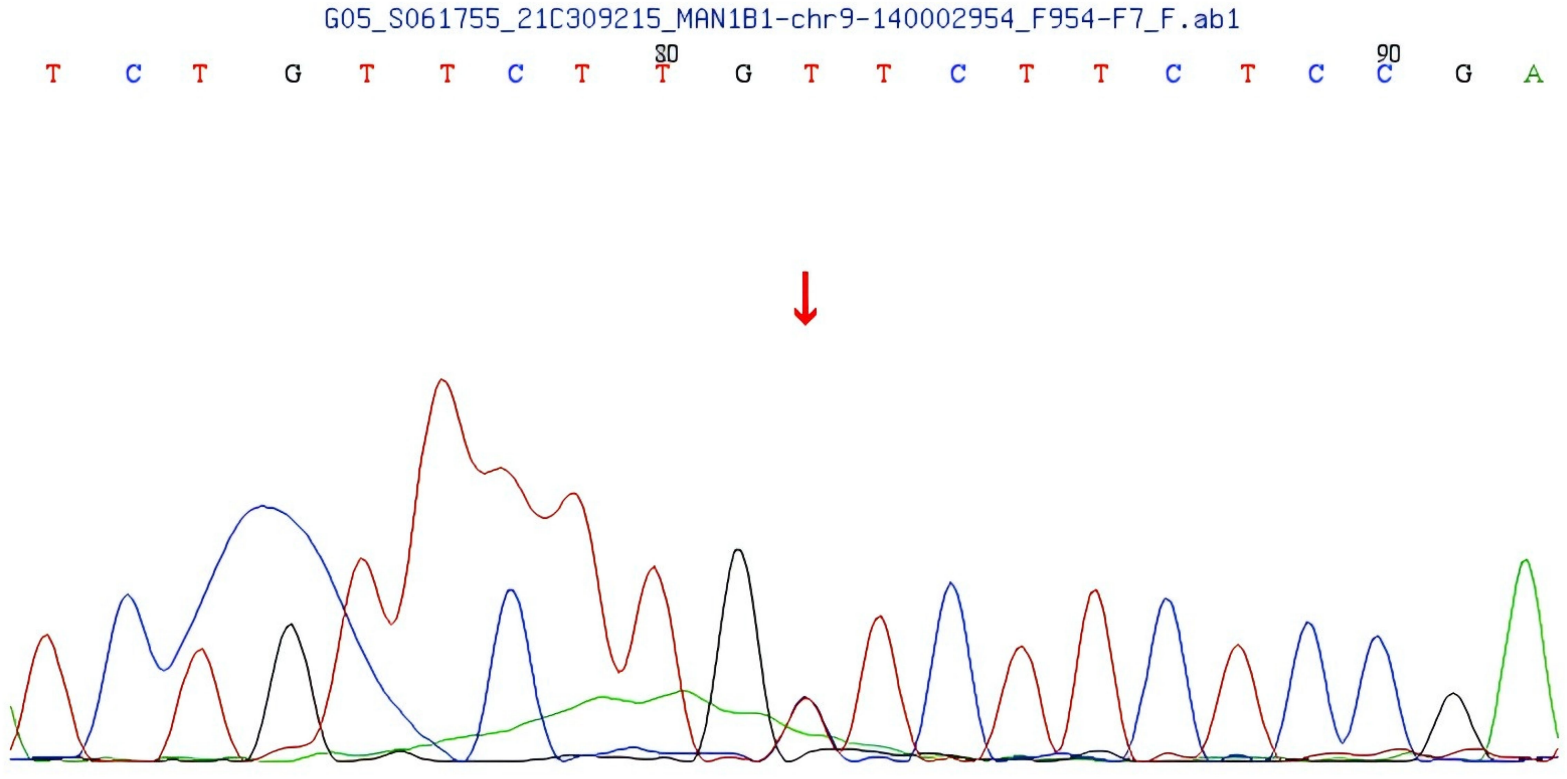
MAN1B1 gene: missense variant c.2011C>T.

The second variant, located in exon 13 of the MAN1B1 gene, is a compound heterozygous missense variant (c.2011C>T), resulting in the p.L671F amino acid substitution (See **Figure 5**). Family pedigree analysis confirmed maternal inheritance of the c.2011C>T compound heterozygous missense variant, while the father had a normal genotype at this locus. This variant is not reported in population frequency databases, suggesting it is a rare variant. A literature search revealed no prior reports, and the ClinVar database lacks pathogenicity data for this variant. However, REVEL predicted this variant to be potentially pathogenic. According to the ACMG guidelines, the variant is preliminarily classified as PM2+PM3 (Trans) with uncertain clinical significance.

Additionally, a c.650A>G missense variant was found in exon 6 of the DUOX2 gene in the patient, resulting in an amino acid change of p.N217S. According to the ACMG guidelines, the variant is preliminarily classified as PM2+PM3 (Trans) with uncertain clinical significance.

## 4 Discussion

Rafiq syndrome is a rare autosomal recessive genetic disorder, classified as a congenital disorder of glycosylation type II (MAN1B1-CDGII) (1). With an extremely low incidence rate, just over 40 cases have been reported worldwide (2). Its main clinical features include delayed intellectual and motor development, distinctive facial features, truncal obesity, and decreased muscle tone. The degree of intellectual disability varies, and scholars have reported that most patients have mild to moderate intellectual disability; Due to delayed motor development and decreased muscle tone, the proband may experience delayed or regressed motor skills at a certain stage. Distinctive facial features include downward-slanting palpebral fissures, thin and sparse eyebrows, long eyelashes, protruding nose, thin upper lip, small chin, large ears, low ear position, and short neck. Other manifestations include seizures, overeating, language delay, autism, and physical aggression (1–4).

This syndrome is caused by mutations in the MAN1B1 gene and was first described in 2011 by Rafiq et al, who reported five families with intellectual disability and distinctive facial features —including four consanguineous Pakistani families and one Iranian family. All 12 probands were found to carry MAN1B1 gene mutations, including a missense mutation (p.Glu397Lys) in three Pakistani families, a homozygous nonsense mutation (p.Trp473)* in another Pakistani family, and a missense mutation (p.Arg334Cys) in the Iranian family.

All probands exhibited intellectual developmental disorder of varying severity and delayed motor development compared to normally developing children. They presented with distinctive facial features to varying degrees, including downward-slanting palpebral fissures, hypertelorism, a long face, flat cheekbones, a short philtrum, thin upper lips, a broad nasal root, and a small chin. At the same time, we found that there are also cases of trunk obesity, overweight, and overeating, and some patients may exhibit verbal or physical aggression. Two probands experienced seizures at the ages of 6 and 10 years, with imaging studies revealing no significant white matter abnormalities or cerebellar atrophy. Based on these findings, this disorder was named Rafiq syndrome (1).

In 2013, Rymen et al. from Belgium reported 7 patients carrying MAN1B1 gene mutations and classified these cases as congenital glycosylation type II disorder (CDG-II), suggesting that MAN1B1 gene mutations may be a potential pathogenic factor (2, 3). In 2014, Scherpenzeel et al. conducted a study on 100 CDG-II children and identified 11 patients carrying MAN1B1 gene mutations. Two of the patients also showed mild coagulation abnormalities, which is considered a newly discovered clinical feature (3, 5). In 2015, Hoffjan found the mutation of MAN1B1 gene in a close relative family in Türkiye, and all three patients carried c.1000C>T (p.Arg334Cys) mutation (6, 7). Rafiq et al. reported this mutation in 2011. In 2025, Zang et al. reported a consanguineous Pakistani family with multiple affected individuals exhibiting mild dysmorphic facial features, developmental delay, and intellectual disability. Whole-exome sequencing led to the identification of a novel MAN1B1 variant (c.772_775del) (8). Our proband has unique facial features (small forehead, wide eye distance, small bilateral eye slits, low nasal bridge, protruding nose, short stature, small chin, large ears, short neck), delayed critical intelligence, trunk obesity, abnormal coagulation function, abnormal electroencephalogram, etc., which are similar to the clinical manifestations of CDG-II caused by MAN1B1 deficiency reported in the literature. **Table 2** summarizes the clinical symptoms of patients and the characteristics associated with Rafiq syndrome. In addition, the proband also had thyroid secretion disorder type 6, which has not been found in other studies.

**Table 2:** Symptom comparison among the proband, other RIFIQ patients, and individuals with thyroid secretion disorder type 6.

Reference	Intellectual and motor developmental delay	Truncal Obesity	Down-slanting palpebral/Thin lateral eyebrows	Small chin/Thin upper lip	Short philtrum	Protruding nose	Seizures	CH
Rafiq,M.A. 2011 (1)	12/12	2/12	5/12	7/12	2/12	7/12	2/12	0/12
Rymen, D. 2013 (3)	7/7	7/7	7/7	–	6/7	7/7	1/7	0/7
Van Scherpenzeel,M. 2014 (4)	12/12	8/12	7/12	6/12	–	7/12	3/12	0/12
Hoffjan, S. 2015 (7)	3/3	3/3	3/3	3/3	3/3	3/3	0/3	0/3
Balasubramanian, M. 2019 (10)	4/4	3/4	4/4	4/4	4/4	4/4	0/4	0/4
Kasapkara, C.S. 2021 (19)	1/1	1/1	1/1	1/1	1/1	1/1	0/1	0/1
Okamoto N.2021 (20)	1/1	0/1	1/1	1/1	1/1	1/1	0/1	0/1
Current case	1/1	1/1	1/1	1/1	1/1	1/1	0/1	0/1
Thyroid Dyshormonogenesis Type 6	In severe cases, clinical symptoms may manifest.	In severe cases, clinical symptoms may manifest.	–	–	–	–	–	1/1

The MAN1B1 gene (OMIM 604346) is located on chromosome 9q34.3 and consists of 13 coding exons. It is a protein-coding gene that encodes an enzyme belonging to the glycosyl hydrolase family 47, which plays a role in N-glycan biosynthesis. In 2013, MAN1B1 was identified as the first Golgi α-mannosidase associated with human diseases, with this class of enzymes being involved in N-glycan remodeling (9, 10). MAN1B1-CDG is a relatively common subtype of CDG-II, with an autosomal recessive inheritance pattern. Currently, there are no statistical reports on mutation frequency, but over 40 different types of mutations have been reported. In this study, compound heterozygous mutations were identified in the MAN1B1 gene of the patient: c.1281_1303delCATCCACGGCCTGTCTGGGAAGA and c.2011C>T. **Table 3** presents the results of the genetic analysis. Currently, it is unclear whether there is a pattern to follow. The mutations seem to be random, and it is impossible to determine which mutation dominates. However, frameshift mutations were only mentioned once in 2014, and may be even rarer.

**Table 3:** Genetic analysis.

Gene mutation	Reference	Population frequency	Protein prediction
c.1000C>T(p.Arg334Cys) c.1418 G>A(p.Trp473*) c.1189G>A(p.Glu397Lys)	Rafiq, M.A. 2011 (1)	0.001	(ACMG)pathogenic
c.1225T>C(p.S409P) c.172G>T(p.E58X) c.1833_1834delA>G(p.T611del)	Rymen, D. 2013 (3)	Low-frequency variant	(ACMG) pathogenic
c.1001G>C(p.R334C ) c.1849C>T(p.Q617X ) c.1976T>G (p.F659C) c.2065G>T(p.E689X ) c.1225T>C(p.S409P) c.1282delA (p.I428fs*43)	Van Scherpenzeel,M. 2014 (4)	Low-frequency variant	(ACMG) pathogenic
c.1000C>T(p.Arg334Cys)	Hoffjan, S. 2015 (7)	0.0078	(ACMG)pathogenic
c.1311del(p.Leu438fs) c.761764del p.Ile254Thrfs*20 1000C>T(p.Arg334Cys)	Balasubramanian, M. 2019 (10)	Low-frequency variant	–
c.782G>A(p.(Trp271*)	Kasapkara, C.S. 2021 (19)	Low-frequency variant	(ACMG)pathogenic
c.1837del p.Asp613Thrfs*115	Okamoto N 2021 (20)	Low-frequency variant	(ACMG) pathogenic
c.772_ 775del CAGG p. L258Mfs*16	Liyu Zang 2025	Low-frequency variant	(ACMG) pathogenic
c.1281_1303delCATCCACGGCCTGTCTGGGAAGA(p.H427Qfs*33) c.2011C>T(p.L671F)	Ruifang Qi 2025	Low-frequency variant	(ACMG)Likely pathogenic

The pathogenesis of MAN1B1-CDG remains unclear. Current research has confirmed that MAN1B1 is localized in the Golgi apparatus and encodes a Golgi α-mannosidase, which plays a role in N-glycan remodeling. MAN1B1 catalyzes the removal of terminal mannose residues from Man9GlcNAc2 intermediate branches in the Golgi, producing the Man8GlcNAc2 isomer B. This enzyme is thought to be involved in glycoprotein quality control, and its functional deficiency leads to the multisystem disorder MAN1B1-CDG. However, the pathogenesis remains to be further elucidated (11–13).

This proband has similar clinical manifestations. Combined with gene sequencing, a compound heterozygous mutation was discovered in the MAN1B1 gene, diagnosed as Rafiq syndrome. Unlike other Rafiq syndromes, this proband was diagnosed with congenital hypothyroidism at 2 months old and received LT4 treatment. Thyroid ultrasound shows tongue ectopia. Meanwhile, gene sequencing also revealed a missense mutation c.650A>G in exon 6 of the DUOX2 gene. Rafiq syndrome with congenital hypothyroidism, has not been reported yet and is the first discovery in China.

In 2002, Moreno et al. first reported CH caused by DUOX2 gene mutations and named it “Thyroid Hormone Production Disorder Type 6” (14). The DUOX2 gene is located on chromosome 15q15.3, spanning approximately 22kb and consisting of 34 exons. DUOX2 protein is a membrane protein expressed on the apical membrane of thyroid follicular cells. Its C-terminus contains six transmembrane alpha helix structures and one FAD and NADPH binding site, while its N-terminus contains a peroxidase like domain and a transmembrane helix structure. DUOX2 protein acts as a catalytic subunit of calcium dependent NADPH oxidase II (Ca ²/NADPH) to synthesize hydrogen peroxide (H2O2). DUOX2 gene mutation can disrupt the production of H2O2, damage iodine oxidation and tissue, ultimately leading to insufficient synthesis of thyroid hormones. Many international studies have confirmed that loss of function mutations in the DUOX2 genome can prevent the synthesis of H2O2 in the thyroid gland, resulting in CH. So far, the Human Gene Mutation Database (HGMD) has recorded over 80 DUOX2 mutations, including more than 30 different types such as missense mutations, nonsense mutations, splice site mutations, and deletion mutations (15). Most children with congenital hypothyroidism (CH) are asymptomatic at birth and are typically detected through abnormal newborn screening results. Clinical manifestations tend to become more apparent during childhood, often including distinctive facial features and delayed neurodevelopment. Newborn screening in China was first implemented in the 1980s. Over the past four decades, a comprehensive nationwide screening network has been established, facilitating the early identification of an expanding range of genetic, metabolic, and rare disorders in neonates, thereby improving child health outcomes. Newborn screening remains critical for the timely detection of treatable conditions such as congenital hypothyroidism, even in individuals presenting with complex and syndromic phenotypes.

The patient was diagnosed with CH at 2 months of age and received LT4 treatment. At 5 months of age, the five thyroid function tests had not yet returned to normal. At 6 months, the five thyroid function tests were normal. At 1 year and 2 months of age, the patient could walk and speak in simple sentences. At 3 years of age, insufficient LT4 dosage was discovered and adjusted in a timely manner. Except for widened eye distance, low nasal bridge, and delayed intellectual development, no other typical changes of CH were found. We feel that most dysmorphic features can be attributed to the MAN1B1 defect given the strong overlap with previously reported cases of Rafiq syndrome. The thyroid ultrasound of the proband showed no definite thyroid echo detected at the thyroid level, only a hypoechoic nodule with a size of approximately 1.3 × 1.1 × 1.2cm was detected at the base of the tongue, suggesting tongue ectopia. At the same time, a missense heterozygous mutation c.650A>G was found in exon 6 of the DUOX2 gene during gene sequencing of the patient, resulting in an amino acid change p.N217S. There have been reports of tongue ectopic thyroid combined with hypothyroidism (16–18). Studies have demonstrated that mutations in key transcription factors essential for thyroid development and differentiation—such as NKX2-1 (TITF-1), PAX8, HHEX, and FOXA1—can result in aberrant thyroid migration. To date, there is no evidence supporting a causal relationship between DUOX2 gene mutations and ectopic thyroid. We propose that the underlying etiology of congenital hypothyroidism (CH) in the proband is attributable to the combination of a DUOX2 gene mutation and ectopic thyroid, rather than a mutation in the MAN1B1 gene. MAN1B1 functions in protein quality control within the Golgi apparatus, whereas DUOX2 is critically involved in hydrogen peroxide production in the thyroid gland. No shared molecular pathway or direct interaction between these two genes has been identified, and they are not known to participate in the same biological process. In thyroid cells, the Golgi apparatus plays a central role in the post-translational glycosylation of thyroglobulin. Given that both proteins may undergo glycosylation, it remains unknown whether impaired glycoprotein quality control due to MAN1B1 deficiency could indirectly influence the structure or function of DUOX2, a potential glycoprotein. This represents a novel area for future investigation. The urine screening of this proband showed mild keto dicarboxylate urine and slightly high levels of succinic acid. This manifestation has not been reported in other Rafiq syndromes and is considered an incidental phenomenon.

## 5 Prognosis and outcomes

Currently, there is no cure for this syndrome, and treatment remains symptomatic, tailored to the patient’s specific clinical manifestations. In this study, the 9-year-old patient presented with intellectual developmental delay, an obese habitus, hepatic steatosis, and CH, with a weight of 50 kg(>P95), height of 138.5 cm (P50), and BMI of 26.2 kg/m² (>P95). Oral levothyroxine (Euthyrox) at a dosage of 75 μg/day was administered once daily, with routine monitoring of the thyroid function panel (five parameters). A low-fat, low-calorie diet was recommended, with height and weight monitored every 3 months, and growth hormone therapy was initiated as needed to improve final adult height. For developmental delay, rehabilitation therapy was initiated in the rehabilitation department, but the response to therapy was poor. Comprehensive EEG findings indicated abnormalities, but no epileptic seizures have been observed to date. Routine follow-ups with the neurology department were recommended.

## 6 Conclusion

In conclusion, we report a case of Rafiq syndrome co-occurring with thyroid dyshormonogenesis type 6 in a Chinese pediatric patient. The clinical features are consistent with those previously described in the literature for Rafiq syndrome. Genetic analysis revealed compound heterozygous pathogenic variants in the MAN1B1 gene: c.1281_1303delCATCCACGTGTCTGGAAGA (p.His427_Gln433del) and c.2011C>T (p.Arg671*). Additionally, a novel missense variant c.650A>G (p.Tyr217Cys) was identified in exon 6 of the DUOX2 gene. Notably, this patient presents with both thyroid dyshormonogenesis and lingual ectopic thyroid—a dual thyroid abnormality not previously documented in individuals with Rafiq syndrome. These findings represent the first report of such a phenotypic combination, thereby expanding the clinical and molecular spectrum of Rafiq syndrome and highlighting the importance of comprehensive genetic evaluation in patients with complex syndromic presentations.

## Data Availability

The datasets presented in this article are not readily available because of ethical and privacy restrictions. Requests to access the datasets should be directed to the corresponding author.
